# Reconfigurable single-material Peltier effect using magnetic-phase junctions

**DOI:** 10.1038/s41598-021-03754-2

**Published:** 2021-12-20

**Authors:** Kurea Nakagawa, Tomoyuki Yokouchi, Yuki Shiomi

**Affiliations:** Department of Basic Science, The University of Tokyo, Meguro, Tokyo 153-8902 Japan

**Keywords:** Magnetic properties and materials, Electronic and spintronic devices

## Abstract

Peltier effects, which produce a heat flux at the junction of two different materials, have been an important technology for heating and cooling by electrical means. Whereas Peltier devices have advantages such as cleanliness, silence, compactness, flexibility, reliability, and efficiency, relatively complicated modular structures are unavoidable, leading to a higher cost than that of commonly used refrigeration technology. Here, we provide a concept of a Peltier device composed of a single magnetic material exhibiting a first-order magnetic transition. Our concept is based on a controllable junction structure consisting of two magnetic phases with opposite Peltier coefficients instead of a semiconductor junction. Using ${\mathrm{Mn}}_{1.96}{\mathrm{Cr}}_{0.04}\mathrm{Sb}$ samples with the first-order magnetic transition between ferrimagnetic (FI) and antiferromagnetic (AF) states, we successfully made a stable junction structure of AF/FI/AF by a pulse heating method and achieved a maximum Peltier coefficient of 0.58 mV. Our device concept was further verified by a numerical simulation based on a finite element method. The single-material Peltier effect reported here avoids a complex device design involving material junctions and is importantly reconfigurable.

## Introduction

Combining the effects of magnetism, heat, phase change and other effects at the material level can produce diversified functions, which is of great significance for achieving high integration of devices^1–3^. Especially, spin-caloritronic and thermomagnetic effects have great potential for highly efficient thermoelectric conversion with simple device configurations^4,5^. For example, the spin Seebeck effect^6,7^ and anomalous Nernst effect^8^ can be used for simple-shaped thermoelectric generators. Conversely, heat management by a spin current or a magnetization-dependent charge current flowing in magnets has also attracted much attention. For example, a heat current is generated by a pure spin current, which is the reciprocal effect of the spin Seebeck effect, namely, the spin Peltier effect^9,10^.

**Figure 1:**
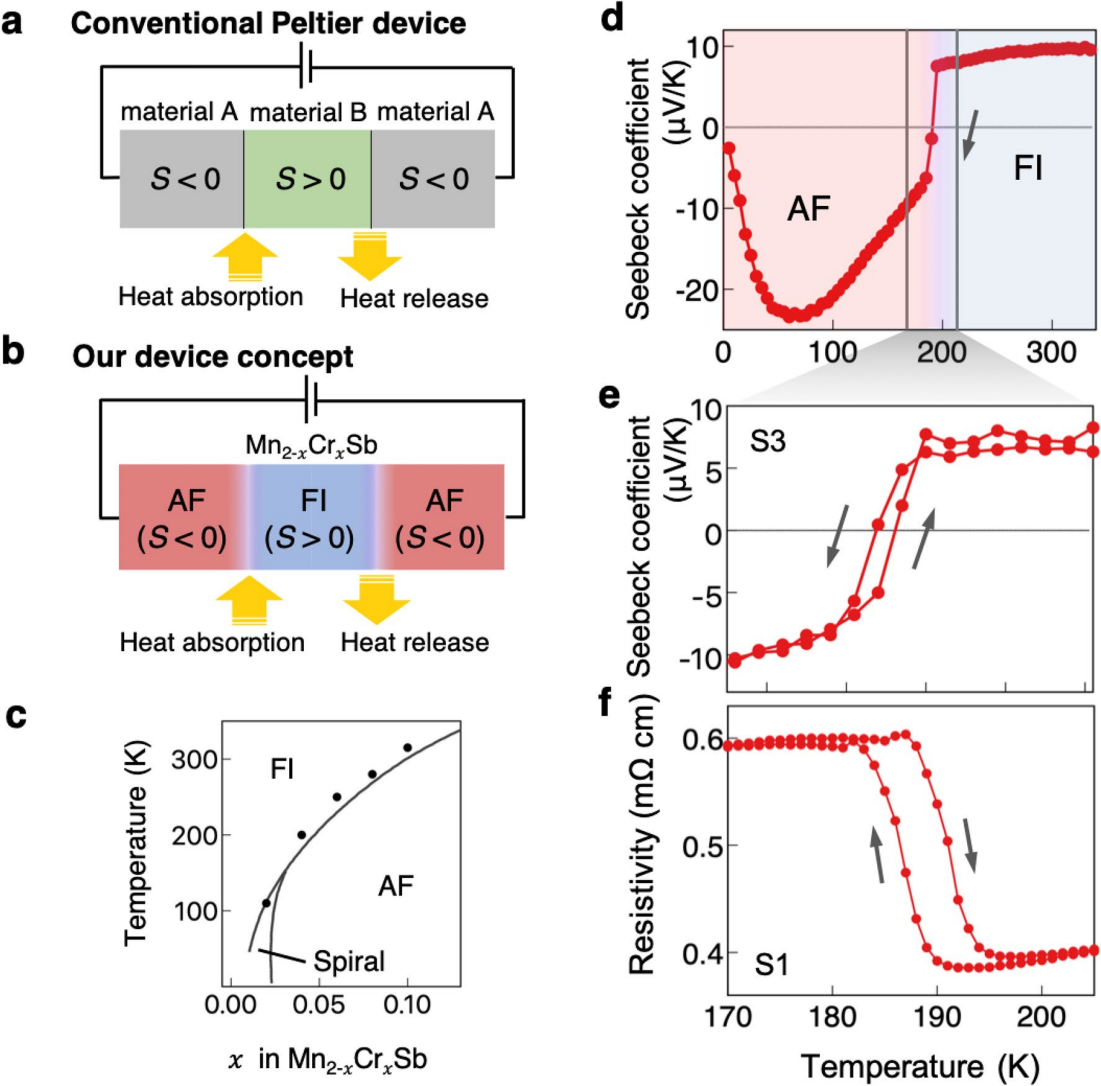
Device concept and sample properties. (**a,b**) Schematics of the conventional Peltier device (**a**) and the single-material Peltier device proposed in this study (**b**). Conventional Peltier devices are composed of two conductors that have Seebeck coefficients of opposite signs; usually, the materials are *p*-type and *n*-type semiconductors. When a charge current flows across the junctions, the Peltier effect induces heat absorption or release at each interface. In the proposed Peltier device (**b**), by contrast, two magnetic phases with different Seebeck coefficients coexist in a single material. When a charge current is applied, heat is absorbed or released at each phase boundary, similar to the conventional Peltier device. (**c**) Magnetic phase diagram of ${\mathrm{Mn}}_{2-x}{\mathrm{Cr}}_{x}\mathrm{Sb}$. Solid curves are phase boundaries reported in the literature^22^. The dots indicate the transition temperatures of our samples, which are determined from the resistivity measurement^25^. (**d)** Temperature dependence of the Seebeck coefficient for a Mn_1.96_Cr_0.04_Sb sample. (**e–f**) Temperature dependence of the Seebeck coefficient (**e**) and the resistivity (**f**) for Mn_1.96_Cr_0.04_Sb around the AF-to-FI transition temperature.

Another example is Peltier cooling and heating by using the anisotropic magneto-Peltier effect^11^, which offers a single-material Peltier device as opposed to the conventional Peltier devices based on a material junction; generally, Peltier cooling and heating devices consist of a junction of two conductors with different Peltier coefficients (${\varPi }_{\mathrm{A}}$ and ${\varPi }_{\mathrm{B}}$). The Peltier coefficient describes the amount of heat carried by a unit charge current^12^. When a charge current (*J*) flows across the junction, heat is generated or absorbed at the interface (Fig. 1a). Here, the heat density absorbed at the interface per unit time $\dot{Q}$ is proportional to the difference between the two Peltier coefficients as follows: $\dot{Q} =\left({\varPi }_{\mathrm{A}}- {\varPi }_{\mathrm{B}}\right)J={\varPi }_{\mathrm{A}/B}J$, where ${\varPi }_{\mathrm{A}/B}$ is the Peltier coefficient of the junction structure. The conventional Peltier devices consist of a junction of *p*- and *n*-type semiconductors^13^, in which, however, the heat exchange point cannot be changed freely once the device is created. Recently, a reconfigurable Peltier device has been demonstrated with the use of the anisotropic magneto-Peltier effect^11^, in which the Peltier coefficient depends on the magnetization direction with respect to the heat current direction. Because of this effect, a local change in the magnetization direction brings about a local change in the Peltier coefficient, and thus, a junction of regions with different magnetization directions acts as a Peltier device^11^. Although this approach offers a Peltier device with high controllability and flexibility, a special shape or a local external magnetic field is required to change the magnetization direction locally.

Here, we provide another way to realize a single-material Peltier device that requires neither a special shape nor an external magnetic field. Our approach is based on a phase coexistence due to the first-order transition nature of two magnetic states whose signs of the Peltier coefficients are opposite to each other; in several materials with a first-order magnetic transition, the signs of the Peltier coefficients [or equally the signs of the Seebeck coefficients (*S*) because of the relationship of $\varPi$ and *S*, $\varPi =ST$ ^14^] in each phase are opposite due to coupling between the magnetic and electric structures^15–17^. In such a system, since one magnetic state can coexist with the other near the transition temperature, a region with *S* > 0 can be sandwiched by regions with *S* < 0 (Fig. 1b). This structure is similar to that of conventional Peltier devices (Fig. 1a), in which heat is absorbed when an electric current flows from the region with *S* < 0 to *S* > 0 and released when a current flows from the region with *S* > 0 to *S* < 0. In particular, since the metastable state can be easily created, annihilated, and driven by local heating^18,19^, magneto-optical effects^20,21^, and spin transfer/orbit torque^22,23^, the single-material Peltier device proposed here is highly reconfigurable and controllable, which is difficult to realize in conventional Peltier devices based on p-n junctions and a single-material Peltier device utilizing anisotropic magneto-Peltier effect^8^.

To demonstrate this concept, we use the ${\mathrm{Mn}}_{2-x}{\mathrm{Cr}}_{x}\mathrm{Sb}$ compound, which shows an abrupt change in the Seebeck coefficient at a first-order transition temperature^16,17^. While Mn_2_Sb is a ferrimagnetic metal with a Curie temperature of 550 K, chromium-modified Mn_2_Sb is known to exhibit a first-order transition from a ferrimagnetic (FI) to an antiferromagnetic (AF) state with decreasing temperature. The phase-transition temperature increases with increasing chromium content (Fig. 1c). Note that for the compounds with a very small chromium content ($x$ < 0.035), an intermediate phase with a spiral spin configuration was reported instead of the collinear AF structure below the transition temperature^24,25^. In ${\mathrm{Mn}}_{2-x}{\mathrm{Cr}}_{x}\mathrm{Sb}$, since the magnetic structure strongly couples with the electronic structure^26^, the Seebeck coefficient changes dramatically at the AF-to-FI transition, resulting in the sign of the Seebeck coefficient being positive in the FI state and negative in the AF state. Due to the first-order transition nature between AF and FI, the FI state can coexist with the AF state as a metastable state near the transition temperature, and thus, we can make a junction of magnetic states with opposite signs of the Seebeck coefficients (Fig. 1b) by adequately controlling the magnetic states.

In the following, we make a magnetic-phase junction of AF/FI/AF by using a local heating technique and experimentally and numerically demonstrate a single-material Peltier cooling and heating device. Moreover, the relaxation time of the magnetic-phase junction structure under the metastable state is estimated to be more than 10^4^ years. The present work suggests highly versatile application of the single-material Peltier effect without any external magnetic field or restriction on the device shape and plays an important role in developing thermal management systems based on spin-caloritronics.

## Results

### Thermoelectric properties and the first-order transition nature.

${\text{Mn}}_{2 - x} {\text{Cr}}_{x} {\text{Sb}}$ single-crystalline samples with $x$ = 0.04 were used in this work. The samples were cut into cuboids. The largest plane corresponds to the *a–b* plane of the crystal structure, and transport measurements were carried out in the *a–b* plane. First, we examined the thermoelectric properties to demonstrate the first-order transition of Mn_1.96_Cr_0.04_Sb. Figure 1d shows the temperature dependence of the Seebeck coefficient. In the FI phase, the Seebeck coefficient is positive and slightly decreases with decreasing temperature. It changes abruptly at the FI-to-AF transition temperature (approximately 190 K) and shows a negative value in the AF phase. Figure 1e and f show magnified images of the temperature dependence of the Seebeck coefficient and resistivity around the AF-FI transition temperature, respectively. The resistivity shows clear hysteresis at the AF-FI transition, indicating the first-order transition. The Seebeck coefficient is approximately 8 $\upmu$ V/K above the transition temperature (FI phase), while it is approximately − 7 $\upmu$ V/K immediately below the transition temperature (AF phase), also showing hysteresis.

### Device settings and measurement process.

**Figure 2:**
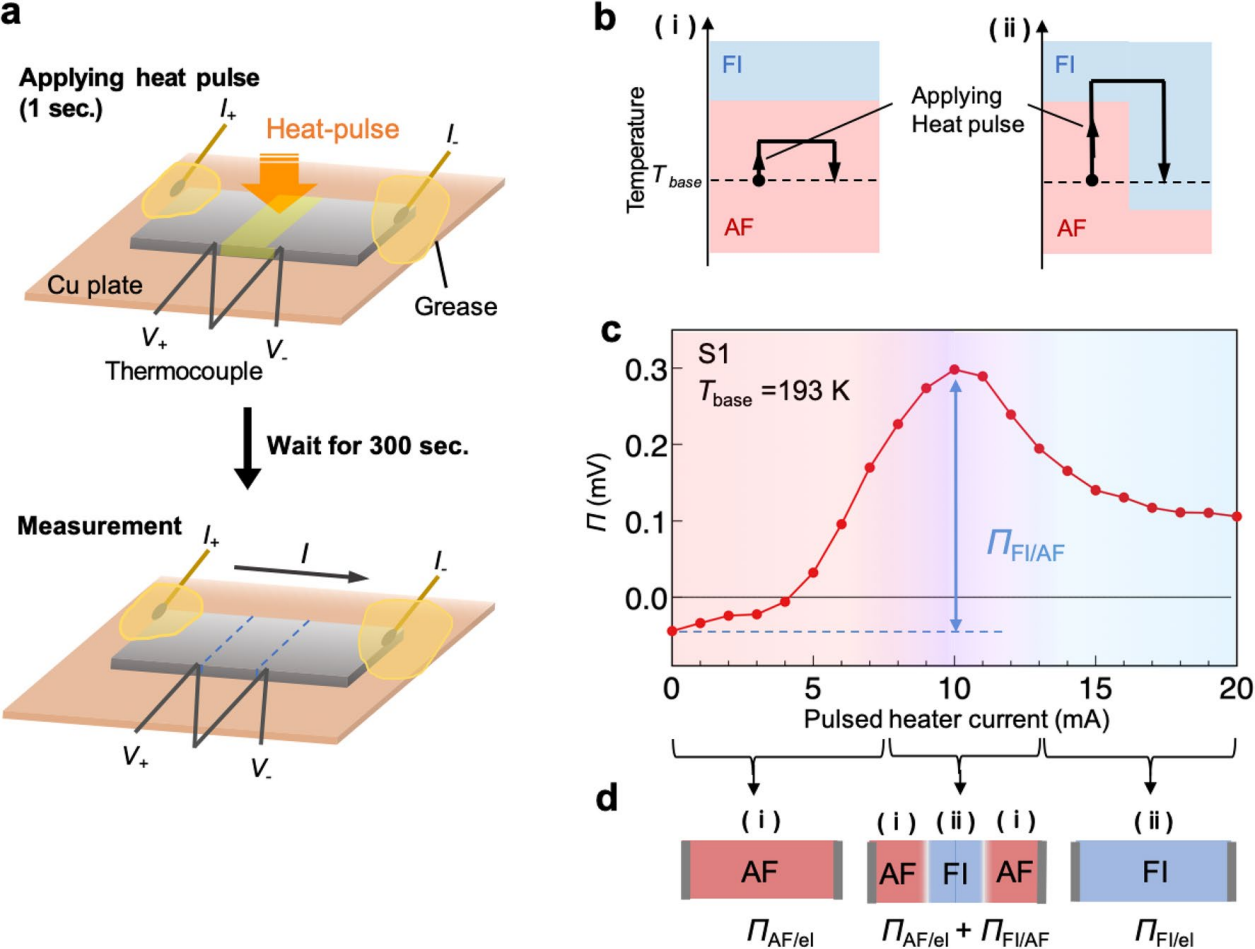
Control of magnetic phases and single-material Peltier effect. (**a**) Schematic of the experimental procedure. We applied a heater current with a pulse width of 1 s to the centre part of the cuboid sample and then waited for 300 s for the sample temperature to return to the base temperature. A charge current was then applied, and the Peltier coefficient was measured (see “Methods” for details). (**b**) Concept of magnetic-phase control using a heat pulse. The vertical axes represent the sample temperature. *T*_base_ is set to lie inside the hysteresis loop. (i) If the pulsed heater current is small, then the final magnetic state remains in the AF phase. (ii) If the pulsed heater current is large enough to make the sample exceed the AF-to-FI transition temperature, then the final magnetic state of the heated part is in the FI phase even after the sample temperature returns to *T*_base_. (**c**) Pulsed heater current dependence of the Peltier coefficient ($\varPi$) observed in the device made of S1. *T*_base_ is set at 193 K. The standard deviations constitute error bars, which are small and invisible in the figure. The red, purple, and blue hatched regions correspond to different magnetic-phase distributions illustrated in (**d**). The single-material Peltier coefficient *Π*_FI/AF_ is defined as the difference between the peak value and the background value at 0 mA (see also **d**). (**d**) Schematics of the magnetic-phase distributions in the device for the three different heater power regions in **c**. The notations (i) and (ii) correspond to the situations described in (**b**).

Our experiment was performed as follows (Fig. 2a and see also Methods for details). First, we lower the sample temperature to 170 K so that the whole sample region is in the AF state as the most thermodynamically stable state. Then, we raise the temperature to the base temperature (*T*_base_). Here, *T*_base_ is set to a temperature within the hysteresis loop (see below for the discussion when *T*_base_ lies outside the hysteresis loop). After that, we supply a heat pulse to the centre part of the cuboid sample by applying a pulsed electric current to a chip heater attached to the centre of the sample (Fig. 2a). This heater current is used to make an AF/FI/AF junction structure; as shown in Fig. 2b, when the pulsed heater current is small, the temperature of the centre region does not reach the AF-to-FI transition temperature. Thus, the whole sample remains in the AF phase [Fig. 2b (i)]. In contrast, when the pulsed heater current is large enough to make the temperature exceed the AF-to-FI transition temperature, the magnetic state of the centre region changes from the AF to FI phase. After turning off the heater current (i.e., the sample returns to *T*_base_), the FI state remains because *T*_base_ is in the hysteresis loop [Fig. 2b (ii)]. In this way, an AF/FI/AF junction structure is created.

### Observation of the single-material Peltier effect

In Fig. 2c, we plot the pulsed heater current dependence of the Peltier coefficient for the device (*Π*) of S1 (see “Methods” for the calculation of *Π*). *T*_base_ is 193 K (inside the hysteresis loop). Without a heat pulse (0 mA), *Π* takes a small negative value. As the pulsed heater current increases, *Π* dramatically increases and reaches its maximum at approximately 10 mA. This large enhancement of *Π* emerges from the creation of the AF/FI/AF structure due to the local heating, as described above (Fig. 2d). This is further confirmed by a numerical simulation (see below). With a further increase in the pulsed heater current from 10 mA, *Π* decreases and almost saturates at approximately 20 mA. The behaviour of *Π* above 10 mA is due to expansion of the FI region; with increasing heater current from 10 mA, the FI region expands towards both sample edges, exceeding the positions where the thermocouple is attached, and finally, the whole sample is in the FI phase at approximately 20 mA. This process induces the observed reduction and saturation of *Π*.

We note that the nonzero *Π* observed in the low- and high-power regions originates from the difference in Seebeck coefficients between the electrodes and the sample; in the case of a low heat pulse, the whole sample is in the AF phase (Fig. 2d). In this situation, absorption and release of heat occur at the interfaces between the Cu electrodes and the AF phase of the sample. Since the Seebeck coefficient of the Cu electrodes is almost 0 $\upmu$ V/K^27^, whereas that of the AF phase is negative, the heat absorption and release at the electrode-AF phase interface is expected to cause a negative Peltier coefficient (*Π*_AF/el_), which is consistent with the sign of the observed Peltier coefficient in the low-power region. This assumption is also justified by our simulation (see below). The positive Peltier coefficient observed in the high-power region also originates from the difference in the Seebeck coefficients between the electrodes and the FI state; since the Seebeck coefficient in the FI state is positive, the observation of a positive Peltier coefficient (*Π*_FI/el_) is consistent with this assumption.

We identified that the peak of the Peltier coefficient at approximately 10 mA corresponds to the state in which the interfaces of the AF/FI/AF junction are located at the positions of the thermocouple. Hence, we define the difference between the peak value and the background value at 0 mA as the single-material Peltier coefficient arising from the AF/FI/AF structure (*Π*_FI/AF_). The maximum value of *Π*_FI/AF_ in the device made of S1 is 0.3 mV, which is comparable to or even higher than the previously reported single-material Peltier coefficient originating from the anisotropic magneto-Peltier effect in Ni (0.11 mV)^11^. A similar dependence of the Peltier coefficient on the pulsed heater current is observed for another device made of S2 (see Supplementary Fig. 2). To confirm the formation of the AF/FI/AF junction structure, we carried out resistivity and magneto-optical Kerr effect (MOKE) measurements. The resistivity of the centre part of the Peltier device decreases after the pulse heating, verifying the formation of the FI domains (see Supplementary Fig. 4 and Note 1). In addition, we observed the formation of the magnetic-phase boundary after the pulse heating using a MOKE technique (see Supplementary Fig. 5 and Note 2).

### Simulation of the single-material Peltier effect

**Figure 3:**
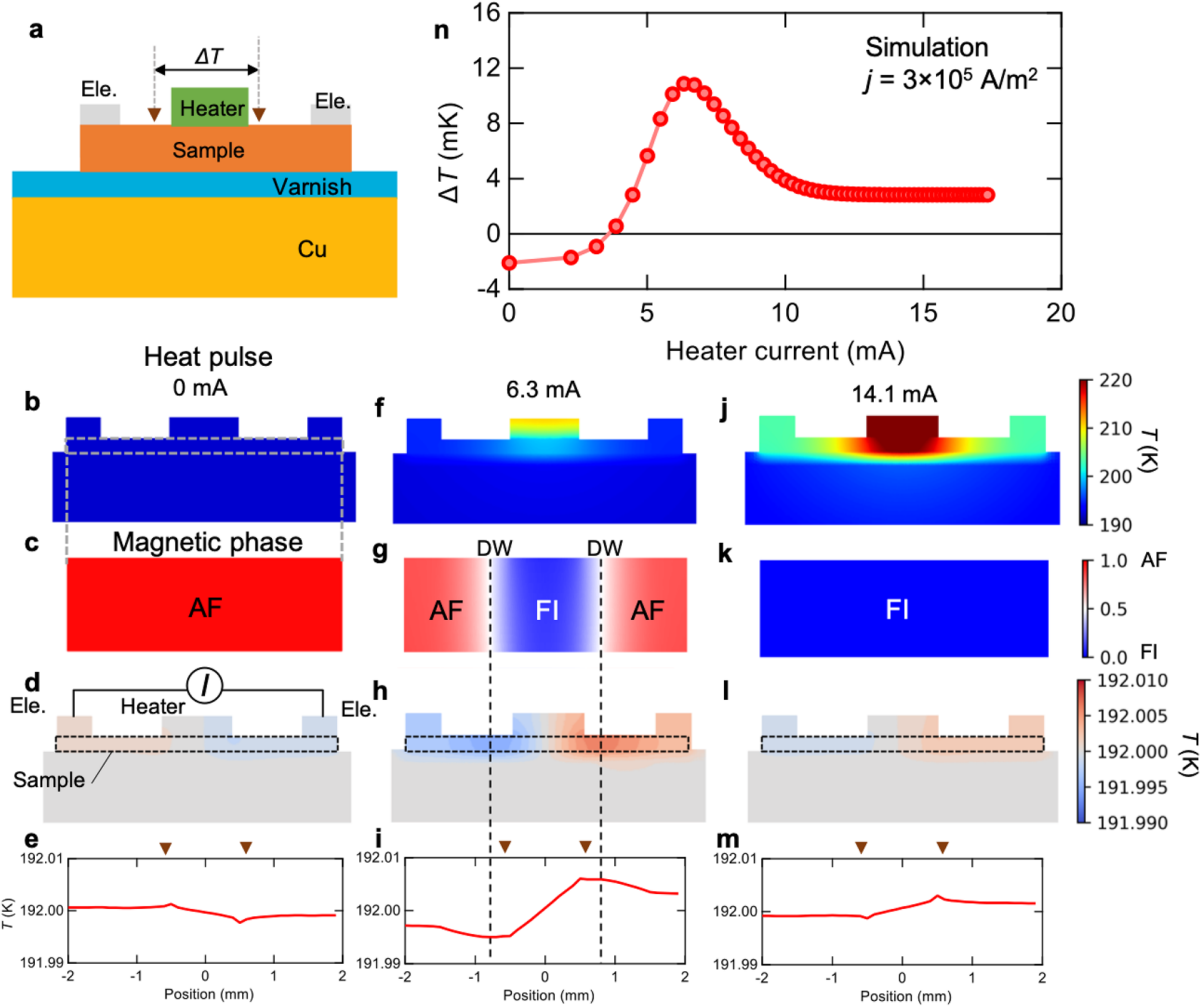
Numerical simulation of the single-material Peltier effect. (**a**) Schematic of the sample configuration in the simulation. (**b–m**) Temperature distributions for pulsed heater currents of 0, 6.3, and 14.1 mA (**b**, **f**, and **j**, respectively), distributions of the magnetic phases derived from the temperature distribution after pulse heating (**c**, **g**, and **k**), temperature distributions originating from the Peltier effect (**d**, **h**, and **l**), and their line profiles plotted as a function of the position (**e**, **i**, and **m**). (**n**) Pulsed heater current dependence of the temperature difference Δ*T* arising from the Peltier effect. Δ*T* was measured between the two points denoted by the inverse triangles in (**a**, **e**, **i**, and **m)**. The electric current density applied for the Peltier measurement was *J* = 3 × 10^5^ A/m^2^.

To further verify that the observed signal arises from the Peltier effect in the AF/FI/AF structure, we performed a simulation based on two-dimensional finite element methods (see “Methods” for details). The model system for this simulation is shown in Fig. 3a. We set the base temperature to 192 K and assumed that the AF-to-FI transition temperature during the heating process is 196 K. At 0 mA, the whole sample is in the AF phase (Fig. 3b,c). The temperature distribution originating from the Peltier effect is shown in Fig. 3d and e. In this case, heat absorption and release occur only at the interfaces between the electrodes and the sample, generating a negative temperature gradient, i.e., a negative Peltier coefficient. When a heater current of 6.3 mA is applied, the temperature in the centre of the sample increases (Fig. 3f). Then, from the temperature increase presented in Fig. 3f, we calculated the distribution of the magnetic phases using the AF-to-FI transition temperature and obtained the AF/FI/AF junction as shown in Fig. 3g (see “Methods” for details). In this case, a large amount of heat is absorbed and released at the AF-FI domain boundary, and the Peltier coefficient becomes positive (Fig. 3h,i). At 14.1 mA, the sample temperature is far above the AF-to-FI transition temperature in the whole sample (Fig. 3j); thus, a single domain FI phase is formed (Fig. 3k). As shown in Fig. 3l and m, heat absorption and release occur only at the interfaces between the electrodes and the sample, bringing about a positive temperature gradient (i.e., a positive Peltier coefficient). These features are quite consistent with the experimental results and the abovementioned explanation. To further confirm the agreement between the experiment and the simulation, in Fig. 3n, we show the heater current dependence of the temperature difference at two points denoted by the triangles in Figs. 3a,e,i, and m. These points correspond to the positions of the thermocouple in the experiment. The profile shows good agreement with the experimental result presented in Fig. 2c. The simulated Peltier coefficient is estimated to be *Π*_FI/AF_ = 0.32 mV, which is the same order of magnitude as the experimental value of 0.3 mV in S1. This consistency indicates that magnetic-phase junctions were indeed realized in our experiment.

### Base temperature dependence of the single-material Peltier effect

**Figure 4:**
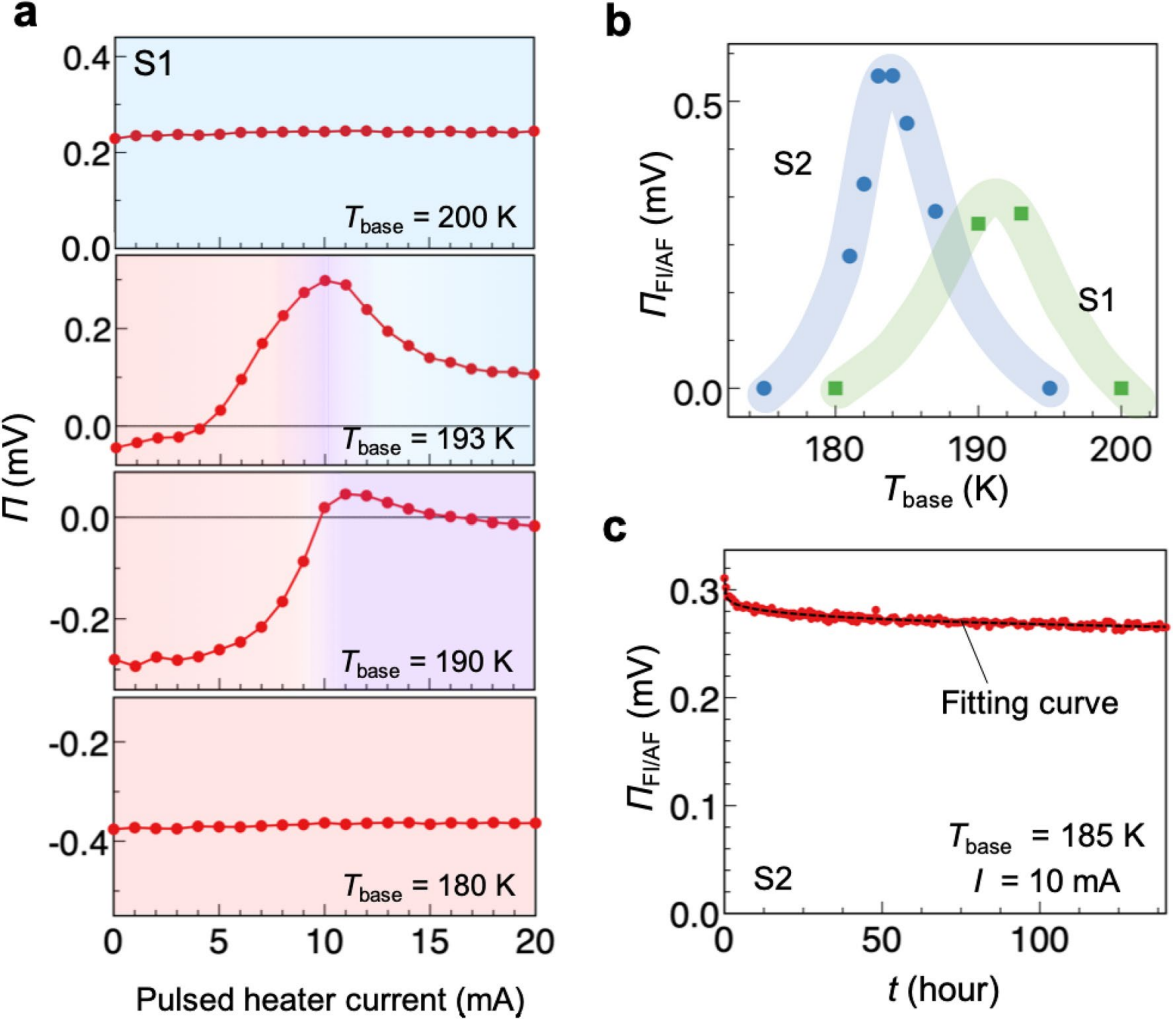
Base-temperature and time dependences of the single-material Peltier effect. (**a**) Pulsed heater current dependence of the Peltier coefficient of S1 measured at *T*_base_ = 200, 193, 190, and 180 K. The red, purple, and blue regions correspond to the different sample situations illustrated in Fig. 2c and d. The standard deviations constitute error bars, which are small and invisible in the figure. (**b**) Base temperature dependence of *Π*_FI/AF_ for S1 and S2. The blue and green curves are guides for the eyes. Error bars are small and invisible in the figure. (**c**) Time dependence of *Π*_FI/AF_ measured for S2. *T*_base_ is set at 185 K, and the pulsed heater current is 10 mA. The dotted line is the fitting curve (see text).

The dependence of *Π* on the base temperature (*T*_base_) also strongly supports that the observed Peltier effect emerges from the magnetic-phase junction. Figure 4a shows the pulsed heater current dependence of the Peltier coefficient of S1 at various *T*_base_. In S1, the FI-to-AF transition temperature (during the cooling process) is 190 K, and the AF-to-FI transition temperature (during the heating process) is 195 K. At *T*_base_ = 180 K, which is sufficiently below the hysteresis loop, *Π* is independent of the heater current (Fig. 4a). This is because the whole sample remains in the AF phase; the heat pulse with 20 mA is probably not sufficient to make the sample temperature exceed the AF-to-FI transition temperature, or even if the FI state is created, the FI state relaxes to the AF state immediately because *T*_base_ is outside the hysteresis loop and the FI state is energetically unstable. In addition, at *T*_base_ = 200 K, because the whole sample is already in the FI phase, *Π* does not vary with the pulsed heater current. In contrast, when *T*_base_ = 190 K and 193 K, which are inside the hysteresis loop, the Peltier coefficients clearly vary as a function of the pulsed heater current; *Π* of *T*_base_ = 193 K shows a peak around the pulsed heater current of 10 mA, which is consistent with the expected behaviour in the single-material Peltier effect due to the magnetic-phase junction, as mentioned above. The Peltier coefficient *Π* at *T*_base_ = 190 K also shows a peak at approximately 10 mA. However, unlike for *T*_base_ = 193 K, the reduction of the Peltier coefficient when the pulsed heater current further increases from 10 mA is small. This is because the heater current is not sufficient to let the side areas of the sample change to the FI phase due to the lower *T*_base_.

In Fig. 4b, the *Π*_FI/AF_ for S1 and S2 is plotted as a function of the base temperature. The base temperature dependences of *Π*_FI/AF_ for S1 and S2 show similar trends, exhibiting a clear peak around the transition temperature. Note that the transition temperatures are slightly different between the samples due to the slight variation in Cr composition; the hysteresis loops in the resistivity of S1 and S2 are observed at approximately 190 K $\le$ *T* $\le$ 195 K and 185 K $\le$ *T* $\le$ 195 K, respectively. The maximum value of *Π*_FI/AF_ is 0.3 mV for S1 and 0.58 mV for S2. The difference in the peak values of the two samples may result from the difference in the clarity of their first-order transition.

### Relaxation of the single-material Peltier effect

Finally, we investigate the stability of the magnetic-phase junction. The magnitude of the single-material Peltier coefficient will be reduced with time because the metastable magnetic state will gradually relax to the equilibrium state. The relaxation of the single-material Peltier coefficient is described as a stretched exponential function as follows (see “Methods”):

1 $$ \begin{array}{*{20}c} {\frac{{\varPi_{{{\text{FI}}/{\text{AF}}}} \left( t \right)}}{{\varPi_{{{\text{FI}}/{\text{AF}}}} \left( 0 \right)}} = exp\left[ { - \left( {t/\tau } \right)^{\beta } } \right],} \\ \end{array} $$

where *τ* is a characteristic relaxation time and *β* is a phenomenological parameter representing the distribution of *τ*. The measured relaxation curve of the single-material Peltier coefficient is shown in Fig. 4c. We created an AF/FI/AF junction by applying a pulsed heater current of 10 mA and monitored *Π*_FI/AF_ as a function of time. The dotted line is a fitting curve with *τ* = 10^8^ h $\approx$ 10^4^ years and yields *β* = 0.18. This guarantees the high stability of the single-material Peltier effect.

## Discussion

We demonstrate a concept of a single-material Peltier device based on a magnetic-phase junction, in which the metastable magnetic phase is controlled with the use of a heat pulse. The consistency between the experiment and simulation supports the formation of the AF/FI/AF structure and the consequent single-material Peltier effect. The single-material Peltier effect observed here enables thermoelectric cooling/heating in a single material without material junctions (see Supplementary Fig. 6 and Note 3 for discussion about application of our concept to practical thermal management systems). The magnetic-phase junction required in our concept is easily created and controlled with various methods^18–23^ in addition to the heat pulse used here. Note that the similar magnitudes of resistivity and Seebeck coefficients and clear phase transitions were reported in polycrystalline samples^13^, in which lower thermal conductivity of phonons can lead to higher thermoelectric performance. Furthermore, our concept of the single-material Peltier effect can also be demonstrated in other materials with first-order transition of two phases whose Seebeck coefficients are different such as FeRh^12^. Magnetic semiconductors may be promising because of larger Seebeck coefficients than magnetic metals. Hence, the magnitude of the Peltier coefficient is expected to be further improved.

## Methods

### Sample preparation and measurement details

Single-crystalline samples of Mn_1.96_Cr_0.04_Sb were prepared by the same method described in our previous paper^28^. The thermal conductivity was measured by a steady-state method. The temperature gradient was measured with thermocouples under a steady heat current given to a sample using a chip resistance heater. We used an own-made sample holder in PPMS for the measurement. The samples were cut into cuboids with dimensions of 0.29 × 1.6 × 3.5  mm^3^ for S1 and 0.48 × 1.6 × 5.2 mm^3^ for S2. (For the measurement of transport properties and additional supplementary measurements, the samples of S3-S5 with dimensions of 0.27–0.49 × 1.1–2.0 × 2.6–5.5   mm^3^ were used.) The sample was fixed on a copper plate with varnish, which had a high thermal conductivity. The contact points of the sample and electrodes were covered with grease to transfer the heat from the contact points to the plate.

To make the magnetic-phase junctions, we supplied a heater current for 1 s, turned off the current, and then waited for 300 s so that the sample returned to the steady state at *T* = *T*_base_. Then, we applied a charge current in the longest direction of the cuboid sample and measured the temperature difference of the centre part of the sample with type-E thermocouples (see also Supplementary Fig. 3 for the accuracy of the measurement of the temperature difference). The measurements were performed three times and averaged. The standard deviations constitute error bars in figures. To remove the extrinsic contribution from Joule heating, we measured the voltage of the thermocouple for both positive and negative current polarities (+ *J* and −*J*). Since the Peltier coefficient is proportional to the current density *J* while Joule heating is proportional to *J*^*2*^ ^29^, we anti-symmetrized the observed voltage as follows: *V* = (V_+*J*_−*V*_−*J*_)/2, where V_+*J*_ and *V*_−*J*_ are the voltages of the thermocouple for positive and negative current polarities, respectively. Then, the temperature difference (Δ*T*) was calculated as follows: Δ*T* = *V/S*_TC,_ where *S*_TC_ is the Seebeck coefficient of the thermocouple. The Peltier coefficient *Π* was calculated as the slope of Δ*T* with respect to the current, assuming the following relationship: $\varPi = -\kappa\Delta T / Jl$. Here, *κ* and $l$ denote the thermal conductivity of the sample and the distance between the thermocouples. We note *κ* contains both phonon and electron contributions. Note that the magnitude of the Peltier coefficient changes almost linearly with the electric current (Supplementary Fig. 1). Two different samples, S1 and S2, were used to check the reproducibility.

### Simulation

We performed the simulation based on two-dimensional finite element methods using FreeFem++ ^30^. The model system for this simulation is schematically shown in Fig. 3a. The sizes of the sample, electrode, heater, varnish layer, and Cu block are $4\times 0.2 \,{\mathrm{mm}}^{2}$, $0.5\times 0.3 \,{\mathrm{mm}}^{2}$, $1\times 0.3 \,{\mathrm{mm}}^{2}$, $9\times 0.2 \,{\mathrm{mm}}^{2}$, and $9\times 2.8 \,{\mathrm{mm}}^{2}$, respectively. The material parameters are summarized in Supplementary Table 1. In the simulation, first, we calculated the increase in the sample temperature when a current was applied to the heater by solving the heat diffusion equation given by

2 $$ \begin{array}{*{20}c} {\nabla \left( {\kappa \nabla T} \right) + q = 0 ,} \\ \end{array} $$

where $\kappa$ and *q* are the thermal conductivity and the power density of the heat source, respectively. For the calculation of the power density of the heater, we assumed that the volume of the heater is $1 \times 0.3 \times 2 \;{\text{mm}}^{3}$. As a boundary condition, *T* = 192 K was assumed at the bottom of the Cu block. Then, we calculated the ratio of the AF-to-FI state (*P*_FI/AF_) as follows:

3 $$ \begin{array}{*{20}c} {P_{{{\text{FI}}/{\text{AF}}}} = \frac{1}{{1 + {\text{exp}}\left[ { - \left( {T - T_{{{\text{AF}} - {\text{to}} - {\text{FI}}}} } \right)} \right]}} ,} \\ \end{array} $$

where $T_{{{\text{AF}} - {\text{to}} - {\text{FI}}}}$ is the AF-to-FI transition temperature (during the heating process). Here, *P*_FI/AF_ = 0 represents the fully AF state, and *P*_FI/AF_ = 1 represents the fully FI state. To take into account the inhomogeneity of the real systems and avoid divergence in the simulation process, we assumed that $P_{{{\text{FI}}/{\text{AF}}}}$ is a continuous function with respect to the position and that $P_{{{\text{FI}}/{\text{AF}}}}$ is 0.5 at $T_{{{\text{AF}} - {\text{to}} - {\text{FI}}}}$. We note that a kind of function forms does not affect our conclusion.

In the next step, we calculated the charge current density (***j***) obtained in the AF/FI/AF junction structure by solving the Poisson equation given by $\nabla \left( {\sigma \nabla \phi } \right) = 0$, where $\sigma$ and $\phi$ are the position-dependent conductivity and scaler potential. Here, the distribution of the resistivity ($\rho = 1/\sigma$) of the sample was calculated as follows: $\rho_{{{\text{FI}}}} P_{{{\text{FI}}/{\text{AF}}}} + \rho_{{{\text{AF}}}} \left( {1 - P_{{{\text{FI}}/{\text{AF}}}} { }} \right)$, where $\rho_{{{\text{FI}}}}$ and $\rho_{{{\text{AF}}}}$ are the resistivities in the FI and AF states, respectively. We applied a voltage of 1 mV to the electrodes. The obtained current density is approximately 3 × 10^5^ Am^-2^, which is comparable to the experimental value. Finally, by using the obtained distribution of the current density and the magnetic phases, we calculated the temperature distribution arising from the Peltier effect in the magnetic-phase junction by solving the thermal diffusion equation [Eq. (2)]. In this process, we calculated the heat source (*q*) as follows: $q = - \nabla \cdot {\mathbf{j}}_{{\text{Q}}} = - \nabla \cdot \left( {\varPi {\mathbf{j}}} \right) = - \nabla \cdot \left( {ST{\mathbf{j}}} \right)$, where ${\mathbf{j}}_{{\text{Q}}} = \varPi {\mathbf{j}}$ is the heat current. The Seebeck coefficient of the sample is given by $S = S_{{{\text{FI}}}} P_{{{\text{FI}}/{\text{AF}}}} + S_{{{\text{AF}}}} \left( {1 - P_{{{\text{FI}}/{\text{AF}}}} { }} \right)$, where $S_{{{\text{FI}}}}$ and $S_{{{\text{AF}}}}$ are the Seebeck coefficients in the FI and AF states, respectively.

### Relaxation of the single-material Peltier effect

The magnitude of the single-material Peltier coefficient will be reduced over time because the metastable state will gradually relax to the most thermodynamically stable state. For estimating the relaxation time of the metastable phases, fitting with a standard or stretched exponential function is typically adopted^31,32^. In fact, in ${\text{Mn}}_{2 - x} {\text{Cr}}_{x} {\text{Sb}}$, the relaxation of the metastable state can be described by the stretched exponential function as follows^33,34^:

$$ V\left( t \right)/V\left( 0 \right) = {\text{exp}}\left[ { - \left( {t/\tau } \right)^{\beta } } \right]. $$

Here, $V\left( t \right)$ is the volume of the metastable phase, τ is a characteristic relaxation time, and *β* is a parameter phenomenologically representing the distribution of *τ*. If *β* = 1, then the equation indicates an Arrhenius-type relaxation, which indicates the single relaxation process. Meanwhile, if 0 < *β* < 1, then the system has multiple relaxation processes, and the relaxation time has dispersion^34^. Since the possible relaxation of the single-material Peltier coefficient is expected to be proportional to the relaxation of the metastable state, the relaxation of the single-material Peltier coefficient can also be described by the stretched exponential function: Eq. (1) in the main text.

## Supplementary Information

Supplementary Information.

## Data Availability

The datasets generated and analysed during this study are available from the corresponding authors upon reasonable request.

## Code availability

The corresponding computer codes are available from the corresponding authors upon reasonable request.
