# Supplementary material for: Reconfigurable single-material Peltier effect using magnetic-phase junctions
Source: Sci Rep. 2021 Dec 20;11:24216. doi: 10.1038/s41598-021-03754-2 (PMC8688509; doi:10.1038/s41598-021-03754-2)
Supplement: Supplementary file 1 — Supplementary Information. [file 41598_2021_3754_MOESM1_ESM.docx]

**Supplementary Information**

**Reconfigurable single-material Peltier effect using magnetic-phase junctions**

**Kurea Nakagawa, Tomoyuki Yokouchi, and Yuki Shiomi**


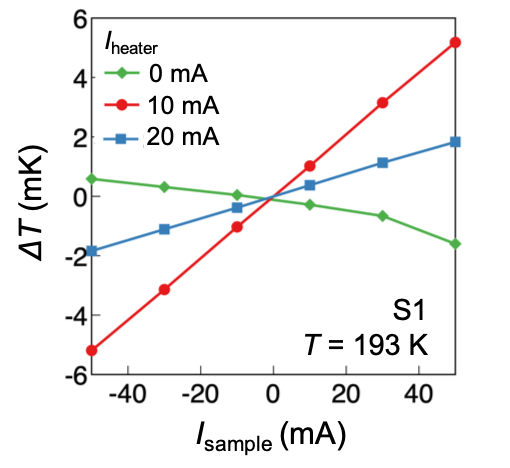


**Supplementary Figure 1:** **Electric current dependence of Peltier signals.** Examples of the observed temperature difference Δ*T* due to the Peltier effect as a function of applied electric current (*I*_sample_). The data is measured for the device made of S1 and *T*_base_ is set at 193 K; this is part of the raw data for the Peltier coefficient shown in Fig. 2c in the main text. We note since we used thermocouples for measuring the temperature differences, Δ*T* is directly obtained (see Methods). The data with the heater current (*I*_heater_) of 0 mA, 10 mA, 20 mA correspond to the situation of “AF/AF/AF”, “AF/FI/AF”, and “FI/FI/FI”, respectively (see also illustrations in Fig. 2d). The standard deviations constitute error bars. We notice that the error bars are small and invisible in the figure. The temperature gradient changes almost linearly with *I*_sample_ for all the heater powers. This means that the contribution from the Joule heating is removed by the anti-symmetrization (see Methods) and the observed temperature gradient is surely due to the Peltier effect. From the slopes of Δ*T*-*I*_sample_ relation, *Π*_FI/AF_ is calculated for each heater current. The similar linear dependence of Δ*T* on *I*_sample_ is observed for different *T*_base_ values and also for S2.


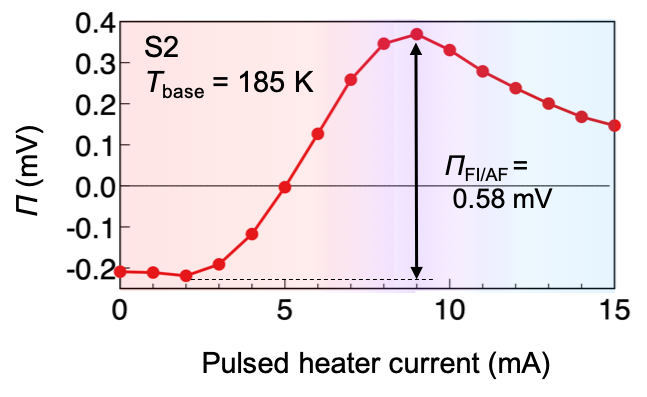


**Supplementary Figure 2: Pulsed heater current dependence of the Peltier coefficient (**$\boldsymbol{\Pi}$**) observed in the device made of S2.** The red, purple, and blue hatched regions correspond to different magnetic-phase distributions illustrated in Fig. 2d in the main text. Similar to the measurement result of S1 shown in Fig. 2c in the main text, the Peltier coefficient of S2 shows clear peak around heater current of 10 mA, which is due to the creation of an AF/FI/AF junction structure. The Peltier coefficient of the AF/FI/AF structure *Π*_FI/AF_ is calculated to be 0.58 mV, which shows the largest magnitude in our experiment. The Peltier coefficient of the AF/FI/AF structure *Π*_FI/AF_ of S2 sample takes maximum value of 0.58 mV at *T*_base_ =185 K.

**
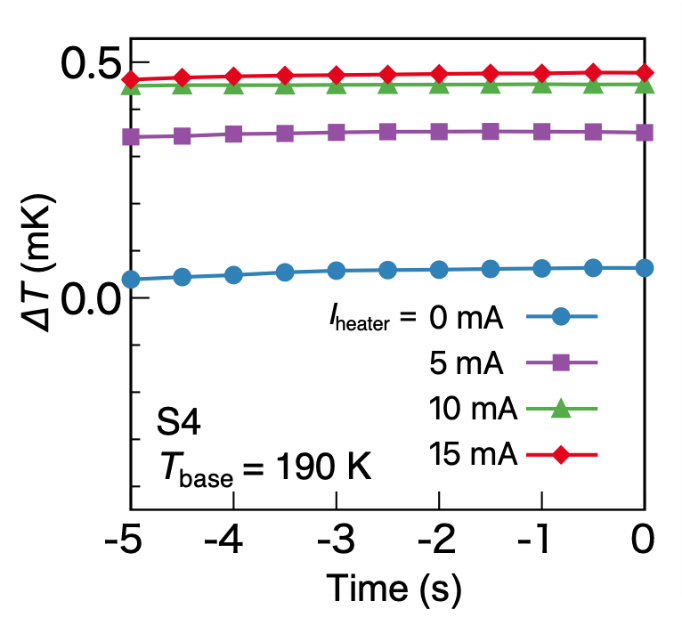
**

**Supplementary Figure 3: Time dependence of temperature differences**. Temperature difference Δ*T* as a function of time just before the measurement of the Peltier effect (i.e, the last 5 seconds in the wait process, see also the Fig. 2 in the main text) for various pulsed heater currents. Here, 0 s corresponds to the time when the Peltier measurement starts. For all pulsed heater current *I*_heater_, temperature differences are steady, supporting the accuracy of the measurement of the temperature difference.

**
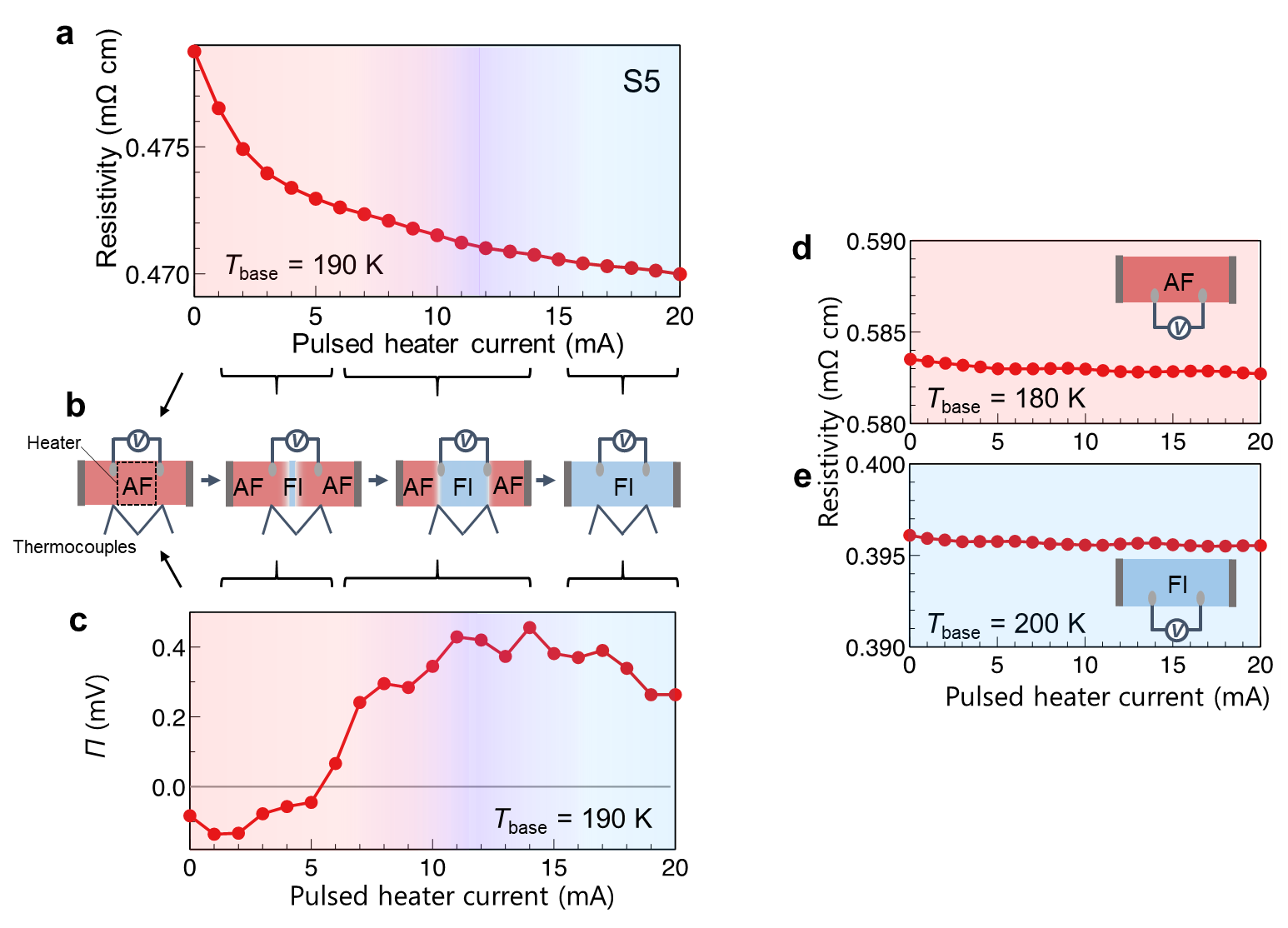
**

**Supplementary Figure 4: Pulsed heater current dependence of the resistivity.** **a** Pulsed heater current dependence of the resistivity at *T*_base_ = 190 K. The setup for the resistivity measurement is illustrated in b. See also Supplementary Note 1. **b** Schematics of the magnetic-phase distributions in the device for different heater currents. **c** Pulsed heater current dependence of the Peltier coefficient (*Π*) measured in the same sample. The pulsed heater current dependence of the Peltier coefficient reported in the main text is reproduced in this sample. **d-e** Pulsed heater current dependence of the resistivity at *T*_base_ = 180 K (d) and 200 K (e). The resistivity is almost constant with heater currents.


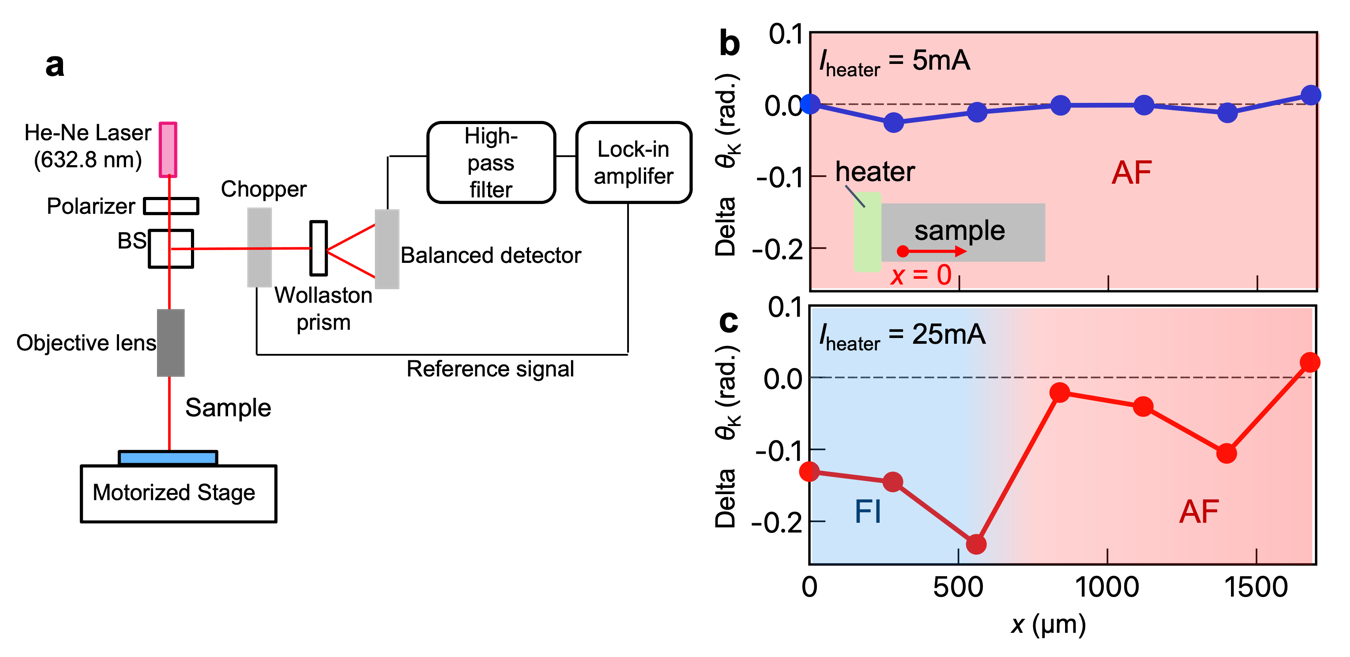


**Supplementary Figure 5: Characterization of magnetic phase junction. a** Schematic diagram of the measurement system for magneto-optical Kerr effect (MOKE). BS denote a beam splitter. See also Supplementary Note 2. **b-c** The background-subtracted Kerr angles (Delta *θ*_K_) at *I*_heater_ = 5 mA (b) and 25 mA (c). Here, the Kerr angle at *I*_heater_ = 0 mA is adopted as the background signal. The origin of the horizontal axis is arbitrarily set to a position near the chip heater and *x* axis is defined as the direction of the arrow in the inset in **b**. The red and blue hatched regions correspond to the AF and FI phases respectively, predicted from the change in the Kerr angles.


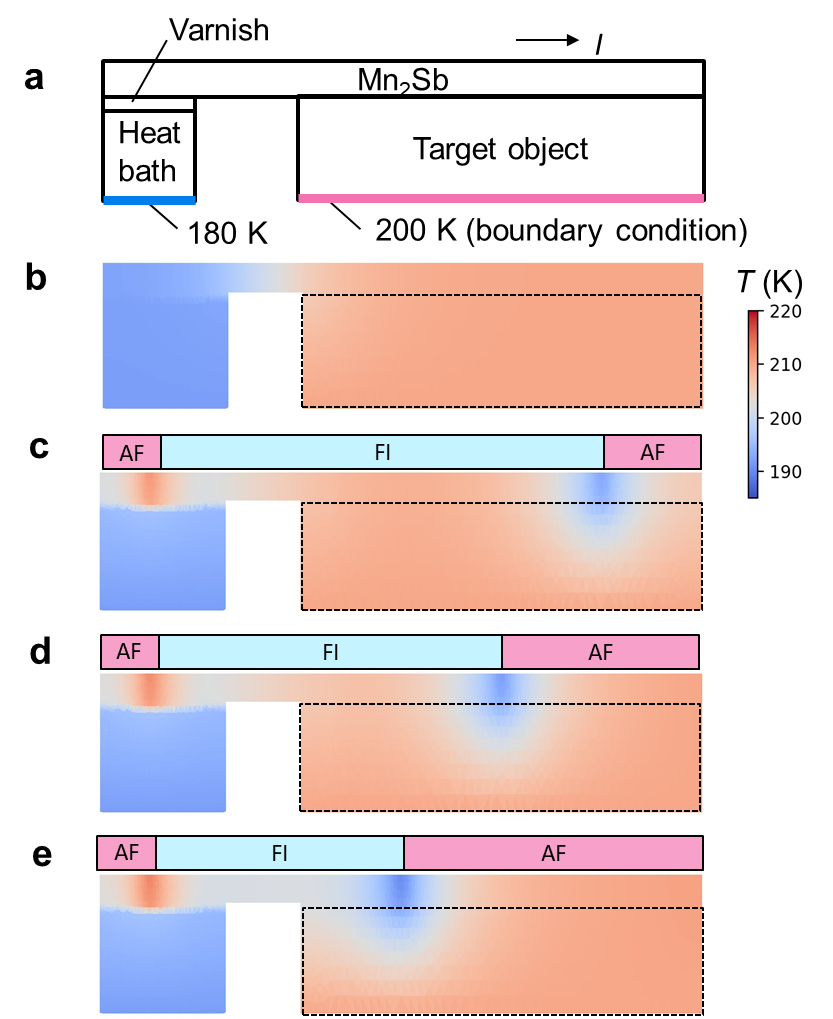


**Supplementary Figure 6: Simulation of reconfigurable Peltier cooling device. a** A schematic of the reconfigurable Peltier cooling system using AF/FI/AF junction structure. One end of the Peltier device is thermally attached to a heat bath, while the other side to the target object. **b, c** Simulation of temperature distribution before applying the current to AF/FI/AF junction (b) and after applying the current to AF/FI/AF junctions with different FI/AF phase boundary positions (c)-(e). As a boundary condition of numerical simulation, the bottom surface of the heat bath is fixed to be 180 K, and target object to be 200 K. We note that the effect of the Joule heating is included in this simulation, and its effect is negligibly small compared to the Peltier effect.

**Supplementary Table 1: Material parameters used for the numerical simulation.**

| Material | | κ(W/mmK) | | *c* (J/gK) | *D* (g/mm^3^) | *ρ* (Ω mm) | *S* (V/K) |
| --- | --- | --- | --- | --- | --- | --- | --- |
| Sample (AF)  Sample (FI) | | 9.1×10^-3^  9.56×10^-3^ | 1.7×10^-2^  5.8×10^-2^ | 7.1×10^-3^  7.1×10^-3^ | 2.8×10^-3^  1.6×10^-3^ | -7×10^-6^  8×10^-6^ |  |
| Electrode (Cu) | | 4.1×10^-1^ | 3.2×10^-1^ | 8.96×10^-3^ | 1×10^-4^ | 1×10^-7^ |  |
| Stage (Cu) | | 4.1×10^-1^ | 3.2×10^-1^ | 8.96×10^-3^ | 1.5×10^-5^ | 1×10^-7^ |  |
| Varnish | | 1×10^-2^ | 3×10^-1^ | 1×10^-2^ | 1×10^8^ | 1 |  |
| Heater | | 1×10^-2^ | 3×10^-1^ | 1×10^-2^ | 1×10^8^ | 1 |  |

*c* and $D$ represent the specific heat capacity and the density, respectively.

**Supplementary Note 1: Pulsed heater current dependence of the resistivity.**

To verify the formation of magnetic-phase junction structure AF/FI/AF, we measured the resistivity of the centre part of the sample. As shown in Supplementary Fig. 4b, we attached the voltage electrodes at both sides of the heater and measured four-terminal resistivity. In this set up, when the FI phase domains are created in the AF phase, the resistivity decreases because the resistivity in the FI phase is smaller than that in the AF phase as shown in Fig.1f in the main text. At *T*_base_ = 190 K (around the AF-to-FI transition temperature), the resistivity decreases as the heater current increases, which indicates the formation of FI domains by local heating technique. In contrast, at *T*_base_ = 180 K, which is sufficiently below the hysteresis loop, the resistivity is independent of the heater current (Supplementary Fig. 4d). This is because the heat pulse is not sufficient to make the sample temperature exceed the AF-to-FI transition temperature. In addition, at *T*_base_ = 200 K, because the whole sample is in the FI phase, the resistivity does not vary with the pulsed heater current (Supplementary Fig. 4e). These results are consistent with the heater current dependence of the Peltier effect (Fig. 4a in the main text). We note that the heater current dependences of resistivity and the Peltier coefficient may be apparently a little different; the heater current at which the resistivity starts to decrease (~1 mA) is smaller than the heater current at which the single-material Peltier effect appears (~4 mA)　(Supplementary Fig. 4c). This difference results from their different dependences on FI magnetic-phase domain formation; the temperature difference due to the Peltier effect is detected when the AF/FI phase boundary reaches the positions of thermocouples, because the heat release and absorption due to the Peltier effect occurs locally at the phase boundary. In contrast, the change in the resistivity is proportional to the fraction of FI phase domains in the whole region between the voltage electrodes, and hence the resistivity decreases even when AF/FI domain boundary lies apart from the voltage electrodes.

In addition, the resistivity change with pulsed heater current at *T*_base_ = 190 K is smaller than the resistivity jump at the transition observed in the *ρ*-*T* measurement (Fig. 1f in the main text). This result suggests that the magnetic phase transition by pulse heating is not induced completely and the phase coexisting state of AF and FI phases is realized. Notably, the observed single-material Peltier coefficient is also smaller than the estimated value from the Seebeck coefficient (*S*) jump at the transition (Fig. 1e in the main text). The ratio of two Δ*S* values is consistent with that of two Δ*ρ* values. which supports the formation of the phase coexistence states. If the perfect AF/FI/AF junction structure is realized, the Peltier coefficient can be enhanced by a factor of 50.

**Supplementary Note 2: Characterization of magnetic phase junction using MOKE.**

In addition to the resistivity measurement, formation of the magnetic-phase junction structure is also confirmed using a magneto-optical Kerr effect (MOKE) (Supplementary Fig. 5). Laser light with the wavelength of λ = 632.8 nm and power of *P* = 5 mW is focused at normal incidence onto the polished surface of the sample using a 50× microscope objective lens. The laser light is modulated by an optical chopper at a frequency of 9.1 kHz. The rotation of the light polarization was measured with the use of a Wollaston prism, a balanced photodetector, and a lock-in amplifier. (see Supplementary Fig. 5a for detailed setup). (Mn,Cr)_2_Sb sample was fixed on a copper plate with varnish and a chip heater was attached to one side of the sample with varnish. Following the procedure of the Peltier effect measurement described in the main text, we first supplied a pulsed heater current, and after the sample returned to the steady state at *T* = *T*_base_, the Kerr rotation angle of the sample surface was measured. The sample used for MOKE measurement is $\mathrm{Mn}_{1.9}\mathrm{Cr}_{0.1}\mathrm{Sb}$, of which the magnetic transition temperature is around 310 K *^2^*, and *T*_base_ was set at 311 K.

In Supplementary Fig. 5b and 5c, we show the background-subtracted Kerr angles at *I*_heater_ = 5 mA and 25 mA (Delta *θ*_K_). Here, the Kerr angle at *I*_heater_ = 0 mA is adopted as the background signal. When *I*_heater_ = 5 mA (Supplementary Fig. 5b), Delta *θ*_K_ is almost zero in the whole measured range, which means that the sample is in the AF state. In contrast, when we apply *I*_heater_ with 25 mA (Supplementary Fig. 5c), the absolute value of Delta *θ*_K_ increases near the heater (small *x* regions), which indicates the existence of the magnetization and thus the formation of FI state. Far from the heater (large *x* regions), the absolute value of Delta *θ*_K_ remains small values, suggesting the AF phase. Hence the FI/AF magnetic phase junction forms with a sufficiently large heater current.

**Supplementary Note 3: Simulation of reconfigurable Peltier cooling device.**

In order to demonstrate that our concept can be applied to an actual thermal management system, we perform a simulation to present an example of device design for a thermal management system utilizing the single-material Peltier effect based on a magnetic-phase junction. The design of the thermal management system is shown in Supplementary Fig. 6a. In this design, a heat sink and a target object are connected by (Mn,Cr)_2_Sb (Supplementary Fig. 6b). When a junction structure of AF/FI/AF is made, the heat is pumped from the target object to heat bath (Supplementary Fig. 6c). This simulation result indicates that our concept is compatible with a practical thermal management system.

In addition, importantly, as described in the main text, one of the advantages of our concept of single-material Peltier effect is controllability and reconfigurability. In other words, the position of cooling part can be changed by manipulating the position of the AF/FI phase boundary, which is difficult in conventional Peltier devices. In fact, our simulation for the practical device design shows that the cooling position can be changed by controlling the position of the AF/FI phase boundary as shown in Supplementary Figs. 6c-e. This result highlights the advantage of practical thermal management systems based on our concept over conventional Peltier devices.

**Supplementary Note 4: Strategy for a wider working temperature.**

In the present study, the working temperature range is limited. For actual applications, working temperature is also important. There are several ways to expand a working temperature range. For example, the phase-transition temperature of (Mn,Cr)_2_Sb monotonically increases with increasing chromium content. Therefore, the device can be designed to work from low temperatures to above room temperature by changing the chromium content (see Fig. 1c in the main text). Furthermore, another approach to increase the working temperature range is the utilization of the quenched technique^1^, which can extend the metastable FI phase to a wider temperature range.

**Reference**

1. F. Kagawa and H. Oike, *Adv. Mat.* **29**, 1601979 (2017).
2. Nakagawa, K. *et al.,* *J. Phys. Soc. Japan* **89**, 124601 (2020).
